# Serum Fusion Transcripts to Assess the Risk of Hepatocellular Carcinoma and the Impact of Cancer Treatment through Machine Learning

**DOI:** 10.1016/j.ajpath.2024.02.017

**Published:** 2024-07

**Authors:** Yan-Ping Yu, Silvia Liu, David Geller, Jian-Hua Luo

**Affiliations:** ∗ Department of Pathology, University of Pittsburgh School of Medicine, Pittsburgh, Pennsylvania; † High Throughput Genome Center, University of Pittsburgh School of Medicine, Pittsburgh, Pennsylvania; ‡ Pittsburgh Liver Research Center, University of Pittsburgh School of Medicine, Pittsburgh, Pennsylvania; § Department of Surgery, University of Pittsburgh School of Medicine, Pittsburgh, Pennsylvania

## Abstract

Hepatocellular carcinoma (HCC) is one of the most fatal malignancies. Early diagnosis of HCC is crucial in reducing the risk for mortality. This study analyzed a panel of nine fusion transcripts in serum samples from 61 patients with HCC and 75 patients with non-HCC conditions, using TaqMan real-time quantitative RT-PCR. Seven of the nine fusions frequently detected in patients with HCC included: *MAN2A1–FER* (100%), *SLC45A2–AMACR* (62.3%), *ZMPSTE24–ZMYM4* (62.3%), *PTEN–NOLC1* (57.4%), *CCNH–C5orf30* (55.7%), *STAMBPL1–FAS* (26.2%), and *PCMTD1–SNTG1* (16.4%). Machine-learning models were constructed based on serum fusion-gene levels to predict HCC in the training cohort, using the leave-one-out cross-validation approach. One machine-learning model, called the four fusion genes logistic regression model (*MAN2A1–FER*≤40, *CCNH–C5orf30*≤38, *SLC45A2–AMACR*≤41, and *PTEN–NOLC1*≤40), produced accuracies of 91.5% and 83.3% in the training and testing cohorts, respectively. When serum α-fetal protein level was incorporated into the machine-learning model, a two fusion gene (*MAN2A1–FER*≤40, *CCNH–C5orf30*≤38) + α-fetal protein logistic regression model was found to generate an accuracy of 94.8% in the training cohort. The same model resulted in 95% accuracy in both the testing and combined cohorts. Cancer treatment was associated with reduced levels of most of the serum fusion transcripts. Serum fusion-gene machine-learning models may serve as important tools in screening for HCC and in monitoring the impact of HCC treatment.

## Graphical abstract

**Figure:**
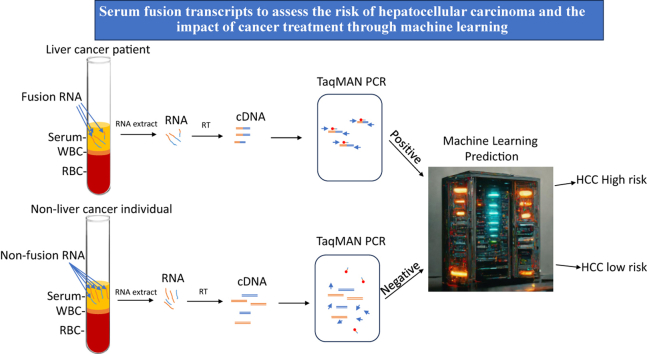

Liver cancer is one of the most fatal malignancies in humans. Worldwide, liver cancer causes >830,000 deaths per year.^1^ Hepatocellular carcinoma (HCC) is the most common type of liver cancer, accounting for 90% of all liver cancers.^1^ In the early stages, liver cancer can be cured through surgical and radiologic intervention. Unfortunately, most liver cancers are insidious and asymptomatic. As a result, over 60% of patients with HCC present in late-stage disease.^2^ The options for treating late-stage HCC are limited. Advanced-stage liver cancer is associated with a high mortality rate. The mean survival time in patients with late-stage HCC is 6 to 20 months.^3^ The 2-year survival rate among patients with late-stage HCC is <50%, while the 5-year survival rate is only 10%. Thus, the identification of early HCC is crucial in reducing the risk for liver cancer–related death.

Currently, serum α-fetal protein (AFP) screening is the most commonly used tool to screen for HCC. However, the accuracy of AFP appears to vary by study, and the cutoff of AFP in the prediction of HCC has ranged widely.^4^^,^^5^ The AFP screening test for HCC tumors measuring <5 cm in diameter has a range of sensitivity of 49% to 71% and a specificity of 49% to 86%.^6^ Thus, a more sensitive tool for early HCC detection is needed.

Recently, a panel of fusion genes was found to be expressed in HCC samples, with variable frequencies.^7^, ^8^, ^9^, ^10^ Four fusion genes, *MAN2A1–FER*, *CCNH–c5orf30*, *PTEN–NOLC1*, and *SLC45A2–AMACR*, expressed with high frequency in liver cancer samples.^7^^,^^8^^,^^11^ The presence of these fusion genes is the result of a chromosome recombination that brings genes from different parts of the genome into juxtaposition with each other. Fusion genes such as *MAN2A1–FER*, *PTEN–NOLC1*, and *SLC45A2–AMACR* are the oncogenic drivers of liver cancer development.^7^^,^^11^^,^^12^ Interestingly, the transcripts of these fusion genes were readily detectable in serum samples from patients with HCC.^8^ These findings suggest a possibility of detecting early HCC by analyzing the fusion transcripts shed into the serum by HCC cells. In this study, nine fusion transcripts in serum samples from 136 individuals were analyzed, with serum fusion transcript detection being predictive of HCC.

## Materials and Methods

### Tissue Samples

The 197 serum specimens and sera used in the study consisted of serum samples obtained from 61 patients with HCC both before and after treatment, and 75 serum samples obtained from individuals without HCC. These samples were obtained from the Pittsburgh Liver Research Center biospecimen core, University of Pittsburgh (Pittsburgh, PA), in compliance with institutional regulatory guidelines. The informed-consent exemptions and protocol were approved by the Institutional Review Board at the University of Pittsburgh. All serum samples were fresh-frozen and stored at −80°C.

### RNA Extraction, cDNA Synthesis, and Detection of Fusion Genes

The procedure was described previously.^13^, ^14^, ^15^, ^16^, ^17^, ^18^, ^19^, ^20^, ^21^, ^22^, ^23^, ^24^, ^25^, ^26^, ^27^, ^28^, ^29^, ^30^, ^31^ Total RNA was extracted using TRIzol to lyse the cancer tissues (InvitroGen, Inc., Carlsbad, CA). First-strand cDNA was synthesized using approximately 5 ng of total RNA from each sample, random hexamers, and Superscript II (InvitroGen) at 42°C for 2 hours. One microliter of each cDNA sample was used for TaqMan PCR reaction, with 50 heat cycles at 94°C for 30 seconds, 61°C for 30 seconds, and 72°C for 30 seconds using primers and probes specific for *MAN2A1–FER* [forward/reverse (F/R), 5′-AGCGCAGTTTGGGATACAGCA-3′/5′-CTTTAATGTGCCCTTATATACTTCACC-3′; TaqMan probe, 5'/56-FAM/TCAGAAAC A/ZEN/GCCTATGAGGGAAATT/3IABkFQ/3′], *SLC45A2–AMACR* (F/R, 5′-TTGATGTCTGCTCCCATCAGG-3′/5′-CAGCTGGAGTTTCTCCATGAC-3′; TaqMan probe, 5'-/56-FAM/AAGAGGGCA/ZEN/TGGTAGTGGAGGC/3IABkFQ/-3′), *CCNH–C5orf30* (F/R, 5′-AAAGTTATTTATCAGAGAGTCTGATGCTG-3′/5′-CTGTTCTACTCCAGGTATTTTCATTATATC-3′; TaqMan probe, 5'-/56-FAM/ACAGGCAAG/ZEN/TTCTGTTCTCTTTCAGCA/3IABkFQ/-3′), *PCMTD1–SNTG1* (F/R, 5′-CTGGAGAGCTTCATCAAAAATAG-3′/5′-CACTTCTCGGGCAATCTCAACA-3′; TaqMan probe, 5′-/56-FAM/AGCTTTGAT/ZEN/AAACTGCTCTCCAGAATGTTG/3IABkFQ/-3′), *ZMPSTE24–ZMYM4* (F/R, 5′-GAGGAAGAAGGGAACAGTGAAGA-3′/5′-CTGGAATAGGGCTCAGTAAAAATGTTATC-3′; TaqMan probe, 5′-/56-FAM/AGACACAGC/ZEN/AGGATGCCAATG/3IABkFQ/-3′), *PTEN–NOLC1* (F/R, 5′-AAGCCAACCGATACTTTTCTCCA-3′/5′-ATAGATGTCTAAGAGGGAAGAGG-3′; TaqMan probe, 5'-/56-FAM/AGACACAGC/ZEN/AGGATGCCAATG/3IABkFQ/-3′), *STAMBPL1–FAS* (F/R, 5′-TTCATCCACACACCAAGGAGC-3′/5′-TGTGCCAGCCTTGTGCACACA-3′; TaqMan probe, 5'-/56-FAM/CAGGCTGTT/ZEN/CAGTATGCTCAG/3IABkFQ/-3′), *VAPB–GNAS* (F/R, 5′-AAGGTGGAGCAGGTCCTGAG-3′/5′-CTCATCTGCTTCACAATGGTGC-3′; TaqMan probe, 5'/56-FAM/TCAAATTCC/ZEN/GAGGTGCTGGAG/3IABkFQ/-3′), *ZNF124–SMYD3* (F/R, 5′-GATGTCGGGACACCCCGGAA-3′/5′-GGCACTGAGAGCATCGCATC-3′; TaqMan probe, 5′-/56-FAM/CAGGCTGTT/ZEN/CAGTATGCTCAG/3IABkFQ/-3′), and β-actin (F/R, 5′-ACCCCACTTCTCTCTAAGGAG-3′/5′-GCAATGCTATCACCTCCCCTG-3′; TaqMan probe, 5'-/56-FAM/CCAGTCCTC/ZEN/TCCCAAGTCCACAC/3IABkFQ/-3′) in a thermocycler (QuantStudio 3; Applied Biosystems Inc., Waltham, MA). A negative control and synthetic positive control were included in each batch of reactions. The PCR products were gel-purified and Sanger-sequenced on 10% positive samples. The threshold determined as positive for fusion transcript was cycle threshold (C_T_) ≤ 45.

### Prediction Model on Fusion-Gene Profile

Machine-learning models were introduced to predict the non-HCC and HCC cases based on the status of the fusion genes. The C_T_ values of nine fusions were measured by TaqMan real-time quantitative RT-PCR and served as the input feature of the machine-learning algorithms. In the training cohort, the associations between individual fusions and non-HCC/HCC status at different C_T_ cutoffs were calculated, and the cutoffs with the highest prediction accuracy, area under the on the receiver operating characteristic curve, and Youden index (sensitivity + specificity – 1) were selected. Then in the multi-fusion models, different fusion combinations with their selected best cutoffs were used as the input feature for the machine-learning model to predict the binomial outcome for the disease status (non-HCC versus HCC). Four machine-learning algorithms were employed, including support vector machine,^32^ random forest,^33^^,^^34^ linear discriminant analysis,^35^ and logistic regression.^36^ Leave-one-out cross-validation (LOOCV) was performed on the training set to select the best model. Finally, the four fusion genes logistic regression model (*MAN2A1–FER*≤40, *CCNH–C5orf30*≤38, *SLC45A2–AMACR*≤41, and *PTEN–NOLC1*≤40) was selected among all of the parameter and algorithm combinations. In the next step, this best model was applied to the testing cohort for performance evaluation. Ultimately, LOOCV was applied to all of the data (pooled training and testing cohorts) to generate the best model for the prediction of new cases in the combined cohort. Biostatistical analysis was performed by statistical software package R version 4.2.2 (*https://cran.r-project.org/bin/windows/base*) with the following packages: randomForest version 4.7-1.1, MASS version 7.3-60.0.1, and e1071 version 1.7-13.

### Prediction Model Integrating Fusion Genes and Serum AFP

When serum AFP was available for the prediction of HCC and benign samples, different AFP cutoffs were applied to generate the receiver operating characteristic curve. In the integration model, the fusion-gene profile was combined with the serum AFP to train the fusion + AFP machine-learning model. When the probability was ≤0.5, it was classified as non-HCC. When the probability was >0.5, it was deemed HCC. Similar to the models involving only fusion-gene data, the models integrating fusion gene + AFP were applied to the training cohort. The best parameters selected by LOOCV were used as the final model for the training cohort and then applied to the testing cohort for evaluation. Lastly, the data from both cohorts were pooled together to provide a final prediction model for the combined cohort. Biostatistical analysis was performed by statistical software package R.

## Results

To analyze the utility of serum fusion transcripts in patients with HCC, a cohort of 136 individuals, including 61 individuals with known HCC and 75 individuals with non-HCC medical conditions, was recruited (Supplemental Tables S1 and S2). All 61 patients with HCC were treated with several types of therapies, including liver transplantation, transarterial chemical embolism, radiofrequency ablation, yttrium 90 isotope radiation, and/or surgical resection (Supplemental Table S1). The serum samples were collected before treatment. To monitor the impact of treatment on the serum fusion-transcript levels, additional samples were collected after treatment. A total of 59% of the HCC cases were Milan-IN status, while 41% were Milan-OUT. The overall mortality rate was 77% (47/61).

### Fusion Transcripts Are Frequently Present in the Serum Samples of Patients with HCC

Nine fusion transcripts were analyzed in the serum samples of 136 individuals. In the pre-treatment serum samples from patients with HCC, the most frequent fusion transcript detected was *MAN2A1–FER* (100%, 61/61) (Table 1), followed by *SLC45A2–AMACR* and *ZMPSTE24–ZMYM4* (both, 62.3%, 38/61). The other frequently detected fusion transcripts in the pre-treatment HCC serum samples were *PTEN–NOLC1* and *CCNH–C5orf30*, accounting for 57.4% (35/61) and 55.7% (34/61) of the total samples, respectively. The relatively low-frequency fusion transcripts were *STAMBPL1–FAS* (26.2%, 16/61), *PCMTD1–SNTG1* (16.4%, 10/61), and *ZNF124–SMYD3* (16.4%, 10/61). The frequencies of these fusion transcripts in the sera of patients with HCC dropped significantly after treatment (Table 1 and Supplemental Table S1). Interestingly, 30.7% (23/75) of individuals without HCC were also positive for *MAN2A1–FER*, albeit mostly in lower quantities (Table 1 and Supplemental Table S2). Other fusion transcripts were also detected in non-HCC individuals, but at relatively low frequencies. The Fisher exact test showed that seven fusion genes—*MAN2A1–FER* (*P* = 1.4 × 10^−19^), *CCNH–C5orf30* (*P* = 1.1 × 10^−5^), *SLC45A2–AMACR* (*P* = 2.8 × 10^−9^), *ZMPSTE24–ZMYM4* (*P* = 9.3 × 10^−6^), *PTEN–NOLC1* (*P* = 1.8 × 10^−6^), *PCMTD1–SNTG1* (*P* = 0.006), and *STAMBPL1–FAS* (*P* = 0.01)—had significantly greater frequencies of expression in the serum samples of patients with HCC, suggesting that the detection of these fusion transcripts in the serum samples of patients with HCC likely predicts the risk for HCC.

**Table 1:** Distribution of Fusion Genes in the Serum Samples

Clinical features	*MAN2A11*-*FER*	*CCNH*-*C5orf30*	*SLC45A2–AMACR*	*PCMTD1–SNTG1*	*ZMPSTE24–ZMYM4*	*PTEN–NOLC1*	*STAMBPL1–FAS*	*VAPB–GNAS*	*ZNF124–SMYD3*
Pre-operation serum fusion positive	100 (61/61)	55.7 (34/61)	62.3 (38/61)	16.4 (10/61)	62.3 (38/61)	57.4 (35/61)	26.2 (16/61)	1.6 (1/61)	16.4 (10/61)
Post-treatment serum fusion positive	39.3 (24/61)	16.4 (10/61)	18 (11/61)	8.2 (5/61)	29.5 (18/61)	24.6 (15/61)	0 (0/61)	0 (0/61)	0 (0/61)
Cirrhosis	100 (60/60)	56.7 (34/60)	63.3 (38/60)	16.7 (10/60)	63.3 (38/60)	56.7 (34/60)	26.7 (16/60)	0 (0/60)	16.7 (10/60)
Ethanol	100 (19/19)	57.9 (11/19)	63.2 (12/19)	10.5 (2/19)	57.9 (11/19)	52.6 (10/19)	36.8 (7/19)	0 (0/19)	15.8 (3/19)
HCV/HBV	100 (28/28)	57.1 (16/28)	67.9 (19/28)	7.1 (2/28)	64.3 (18/28)	57.1 (16/28)	21.4 (6/28)	0 (0/28)	17.9 (5/28)
NASH	100 (19/19)	52.6 (10/19)	63.2 (12/19)	21.1 (4/19)	63.2 (12/19)	57.9 (11/19)	26.3 (5/19)	0 (0/19)	21.1 (4/19)
Milan IN	100 (36/36)	52.8 (19/36)	66.7 (24/36)	16.7 (6/36)	63.9 (23/36)	63.9 (23/36)	27.8 (10/36)	0 (0/36)	16.7 (6/36)
Milan OUT	100 (25/25)	60 (15/25)	56 (14/25)	16 (4/25)	60 (15/25)	48 (12/25)	24 (6/25)	4 (1/25)	16 (4/25)
Well differentiated	100 (15/15)	60 (9/15)	60 (9/15)	13.3 (2/15)	66.7 (10/15)	26.7 (4/15)	33.3 (5/15)	0 (0/15)	0 (0/15)
Well to moderately differentiated	100 (2/2)	50 (1/2)	50 (1/2)	0 (0/2)	100 (2/2)	100 (2/2)	50 (1/2)	0 (0/2)	0 (0/2)
Moderately differentiated	100 (18/18)	72.2 (13/18)	55.6 (10/18)	5.5 (1/18)	66.7 (12/18)	61.1 (11/18)	33.3 (6/18)	0 (0/18)	16.7 (3/18)
Moderately to poorly differentiated	100 (2/2)	0 (0/2)	100 (2/2)	0 (0/2)	100 (2/2)	100 (2/2)	0 (0/2)	0 (0/2)	50 (1/2)
Poorly differentiated	100 (2/2)	0 (0/2)	100 (2/2)	0 (0/2)	50 (1/2)	50 (1/2)	0 (0/2)	0 (0/2)	0 (0/2)
Progress/recurrence	100 (42/42)	52.4 (22/42)	61.9 (26/42)	16.7 (7/42)	54.8 (23/42)	61.9 (26/42)	26.2 (11/42)	2.4 (1/42)	19 (8/42)
Non progress/recurrence	100 (18/18)	61.1 (11/18)	61.1 (11/18)	16.7 (3/18)	66.7 (12/18)	44.4 (8/18)	27.8 (5/18)	0 (0/18)	11.1 (2/18)
Alive	100 (14/14)	35.7 (5/14)	57.1 (8/14)	14.2 (2/14)	64.3 (9/14)	21.4 (3/14)	21.4 (3/14)	0 (0/14)	14.2 (2/14)
Death	100 (47/47)	61.7 (29/47)	38.3 (18/47)	12.8 (6/47)	34 (16/47)	36.2 (17/47)	27.7 (13/47)	2.1 (1/47)	17 (8/47)
Non-HCC individuals	30.7 (23/75)	18.7 (14/75)	13.3 (10/75)	2.7 (2/75)	24 (18/75)	14.7 (11/75)	5.3 (4/75)	4 (3/75)	4 (3/75)

Date are presented as % (*n/N*).

HCV/HBV, hepatitis C virus/hepatitis B virus; NASH, nonalcoholic steatohepatitis.

Association analyses indicated that the presence of *PTEN–NOLC1* in the pre-treatment serum samples was associated with poor survival (*P* = 0.046) and an increased risk for death (*P* = 0.004). Strong expression of *MAN2A1–FER* (≤36 cycles) in the pre-treatment serum samples was also associated with poor survival (*P* = 0.03). The persistent presence of the *MAN2A1–FER* fusion transcript (≤41 cycles, *P* = 0.029) in the serum samples after treatment was associated with a greater risk for less favorable survival outcomes. In addition, the risk for cancer progression/recurrence increased significantly if *CCNH–C5orf30* (*P* = 0.026) or *ZMPSTE24–ZMYM4* (*P* = 0.024) was detected in the serum samples after treatment.

### Prediction of HCC Occurrence by Serum Fusion Transcripts

**Figure 1:**
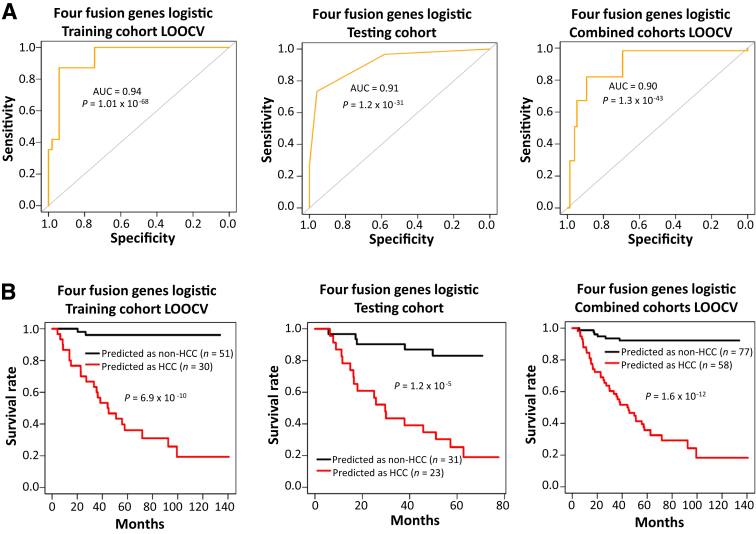
Serum fusion gene model to predict the occurrence of HCC. **A** and **B:** Receiver operating characteristic curve (**A**) and Kaplan-Meier analysis (**B**) of the four fusion genes logistic regression model (*MAN2A1–FER*≤40, *CCNH–C5orf30*≤38, *SL45A2–AMACR*≤41, and *PTEN–NOLC1*≤40) in the training cohort (samples collected before 2016; **A**, left), the testing cohort (samples collected after 2015; **A**, middle), and the combined cohorts (**A**, right). AUC, area under the receiver operating characteristic curve; LOOCV, leave-one-out cross-validation.

To investigate whether serum fusion transcripts predict the occurrence of HCC, samples collected from 2009 to 2015 were utilized as a training cohort, while samples collected from 2016 to 2021 were designated as the testing cohort. There were 82 individuals in the training cohort, including 31 patients with HCC and 51 individuals without HCC. Different combinations of five fusion genes (*MAN2A1–FER*, *CCNH–C5orf30*, *SLC45A2–AMACR*, *ZMPSTE24–ZMYM4*, and *PTEN–NOLC1*) with different cutoff thresholds were used to construct 716 machine-learning models based on the pre-treatment serum fusion-gene data in the training cohort, using LOOCV analysis. A total of 97% (691/716) of these models had a prediction accuracy exceeding 70%, while 56% (404/716) had an accuracy exceeding 80% (Supplemental Table S3). One of these models, called four fusion genes logistic regression (*MAN2A1–FER*≤40, *CCNH–C5orf30*≤38, *SLC45A2–AMACR*≤41, and *PTEN–NOLC1*≤40), reached an accuracy of 91.5%, with a sensitivity of 87.1% and a specificity of 94.1% (Figure 1A and Supplemental Table S3). To test the validity of the model, this algorithm was then applied to the serum samples from the testing cohort, which were collected at a later time and included samples from 30 patients with HCC and 24 non-HCC individuals. The validation test showed that the four fusion genes logistic regression model correctly predicted 83.3% of the statuses of these cases, with a sensitivity of 73.3% and a specificity of 95.8% (Figure 1A and Supplemental Table S4). Combining the training and testing cohorts produced an accuracy of 86% with a sensitivity of 82% and a specificity of 89.3% (Figure 1A and Supplemental Table S5).

Survival analysis showed that 96% (49/51) of individuals survived >10 years when predicted as non-HCC by the four fusion genes logistic regression model in the training cohort, while only 19% (6/31) survived a similar period when predicted as HCC (Figure 1B). In the testing cohort, 84% (26/31) of individuals predicted as non-HCC survived >5 years, while only 17% (4/23) predicted as HCC survived a similar period (Figure 1B). When both cohorts were combined, the same four fusion genes logistic regression model produced a 5-year survival rate of 92% (71/77) in individuals predicted as non-HCC and a 5-year survival rate of 17% (10/58) in individuals predicted as HCC (Figure 1B).

### Incorporation of Serum AFP with Fusion Gene Model

**Figure 2:**
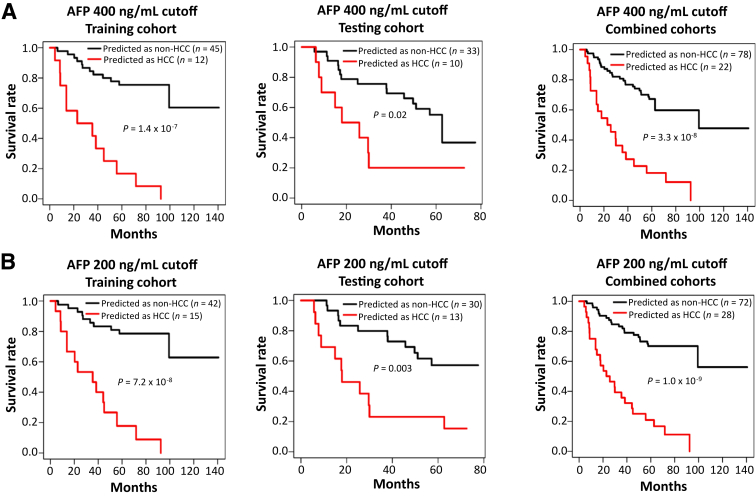
Impact of serum α-fetoprotein (AFP) on the survival of individuals with 400 ng/mL or 200 ng/mL cutoff. **A** and **B:** Kaplan-Meier analysis of serum AFP cutoffs of 400 (**A**) or 200 (**B**) ng/mL in the training cohort (left), the testing cohort (middle), and the combined cohorts (right).

AFP is the primary screening tool for detecting HCC. However, a few non-HCC conditions, including hepatic cirrhosis, may lead to the elevation of AFP. There is a consensus that a level of ≥400 ng/mL may indicate the presence of HCC.^4^ Applying a ≥400 ng/mL AFP cutoff generated an accuracy of 67.2%, with a sensitivity of 38.7% and a specificity of 100% in the training cohort, and an accuracy of 53.5% with a sensitivity of 33.3% and a specificity of 100% in the testing cohort. The overall accuracy of the AFP threshold of ≥400 ng/mL was 61.4% when applied to the combined cohorts. Lowering the threshold of AFP to ≥200 ng/mL^5^ improved the prediction accuracy to 67%, with a sensitivity of 45.9% and specificity of 100%, in the combined cohorts. No individuals with an AFP of ≥400 or ≥200 ng/mL survived through 100 months (Figure 2), while 47.4% (37/78) of individuals with an AFP of <400 ng/mL and 55.6% (40/72) of individuals with an AFP of <200 ng/mL survived through the similar period. These results suggested that high serum AFP had a high specificity in predicting the occurrence of HCC and may complement the fusion-gene model.

**Figure 3:**
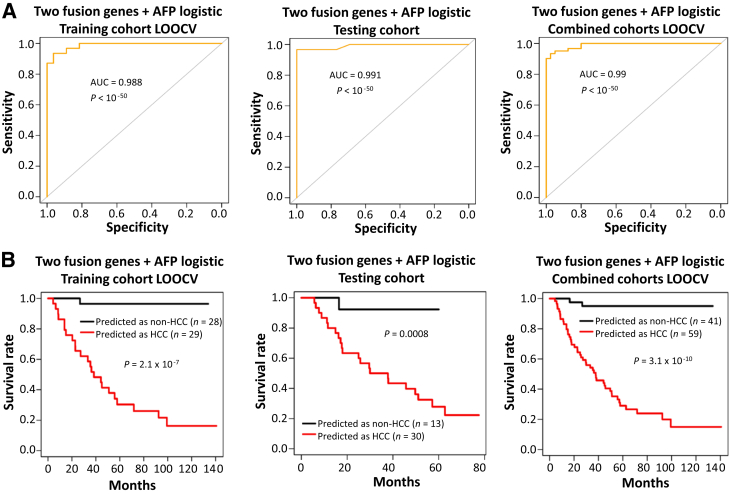
Serum fusion genes and serum α-fetoprotein (AFP) integration model to predict the occurrence of HCC. **A** and **B:** Receiver operating characteristic curve (**A**) and Kaplan-Meier analysis (**B**) of the two fusion genes (*MAN2A1–FER*≤40, *CCNH–C5orf30*≤38) + AFP model in the training cohort (samples collected before 2016; left), the testing cohort (samples collected after 2015; middle), and the combined cohorts (right). AUC, area under the receiver operating characteristic curve; LOOCV, leave-one-out cross-validation.

To investigate the impact of serum AFP level on the fusion-gene model, 716 fusion-gene models were incorporated with serum AFP elements. LOOCV was performed on the serum samples from the training cohort. As shown in Supplemental Table S6, 632 of these models had prediction accuracies exceeding 80% in the training cohort, while 101 of these models had accuracies exceeding 90%. One of these models, called the two fusion gene (*MAN2A1–FER*≤40, *CCNH–C5orf30*≤38) *+ AFP* logistic regression model, had an accuracy of 94.8%, with a sensitivity of 93.5% and a specificity of 96.3% (Figure 3A and Supplemental Table S6). Applying this algorithm to the testing data set resulted in similar accuracy: 95% accuracy, with a sensitivity of 96.7% and a specificity of 92.3% (Supplemental Table S7 and Figure 3A). On combining both cohorts, the two fusion gene + AFP logistic regression model produced an accuracy of 95%, a sensitivity of 95.1%, and a specificity of 95% (Supplemental Table S8 and Figure 3A). The similar accuracies of this model throughout these cohorts suggest that the model is consistent and stable.

Of those individuals predicted as non-HCC by the two fusion gene + AFP model in the training cohort, 96% (27/28) experienced >10 years of survival (Figure 3B). On the other hand, of those predicted as HCC, only 17% (5/29) experienced a similar period of survival. In the testing set, individuals predicted as non-HCC had a 5-year survival rate of 92.3% (12/13), while individuals predicted as HCC had a 5-year survival rate of 23% (7/30) (Figure 3B). In the combined cohorts, individuals predicted as non-HCC had a survival rate of 95% for at least 5 years. Meanwhile, those individuals predicted as HCC had a survival rate of 15.2% for a similar period (Figure 3B). These results suggest that the two fusion gene + AFP logistic regression model was effective in predicting the survival related to HCC.

### Impact of Treatment on the Serum Level of Fusion Transcripts

To examine the potential utility of serum fusion transcripts in monitoring the effects of treatment and the progression of HCC, the post-treatment serum fusion transcripts were analyzed in patients with HCC who had recently undergone treatment for cancer to assess the impact of treatment on fusion-transcript levels. Forty-one percent (25/61) of patients with HCC experienced a complete negative conversion of fusion transcripts in their serum samples after the treatment (Supplemental Table S1). Most of the patients, however, experienced a partial reduction in fusion-transcript levels in the post-treatment sera or experienced only negative conversion of some fusion transcripts but not others. Some patients even had the emergence of new fusion transcripts not detected in the serum samples obtained before treatment. The different dynamics of serum transcripts for different fusion genes after treatment suggested significant heterogeneity of the liver cancers in these individuals and may reflect the dynamics of HCC tumor load impacted by treatment.

**Figure 4:**
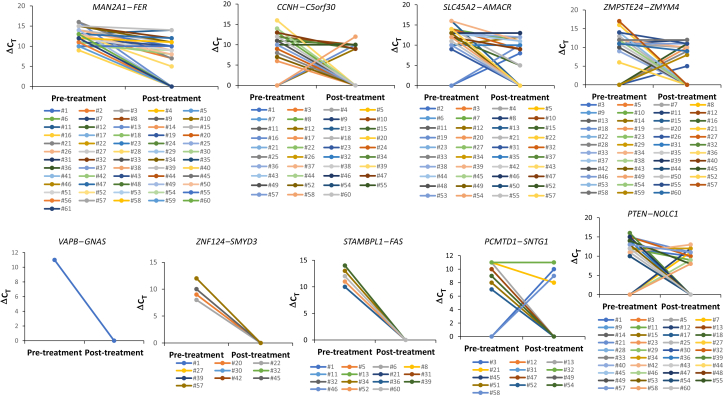
The treatment impact on serum fusion transcript levels. ΔC_T_ was calculated as the difference between the C_T_ of a RT-qPCR of a fusion transcript and the maximal RT-qPCR cycle (50) of a test. Cases with both pre-treatment and post-treatment samples negative for the fusion transcripts were not included in the analysis. The individual cases are indicated in each plot.

As shown in Figure 4, 95% (58/61) of patients with HCC experienced a drop in *MAN2A1–FER* transcript levels in the sera obtained after treatment. Three patients who had no decrease in *MAN2A1–FER* transcript showed distant metastases or HCC progression shortly after treatment (Supplemental Table S1). The treatment of HCC also resulted in decreases in *SLC45A2–AMACR* and *ZMPSTE24–ZMYM4* in 92.1% and 86.8% of the patients with HCC, respectively. Interestingly, in 2 patients with HCC, *SLC45A2–AMACR* emerged as a new transcript in the post-treatment serum, while in 8, *ZMPSTE24–ZMYM4* emerged as a new transcript after treatment. Seven of eight patients with HCC with serum resurgence of *ZMPSTE24–ZMYM4* had either progression or recurrence. The impact of cancer treatments was also found in the serum levels of *CCNH–C5orf30*: 97.1% (33/34) of patients with HCC experienced a drop in the serum levels of the transcript after cancer treatment. However, 5 individuals had *CCNH–C5orf50* emerge as a new transcript in the serum samples after treatment, and all 5 patients with HCC experienced progression, recurrence, or distant metastasis (Supplemental Table S1). When serum transcript dynamics of *CCNH–C5orf50* before and after treatment were used as a factor to assess the risk for HCC progression/recurrence, the serum level drop of less than seven PCR cycles for the *CCNH–C5orf30* transcript in sera after treatment was found to be associated with an increased risk for HCC recurrence/progression (*P* = 0.016). A similar association between *ZMPSTE24–ZMYM4* dynamics and HCC progression was found: A serum level drop of four cycles or less for the *ZMPSTE24–ZMYM4* transcript in sera after treatment signaled an increased risk for HCC recurrence/progression (*P* = 0.03).

## Discussion

All five fusion genes in the prediction models resulted from the underlying chromosome abnormalities in the cancer genome.^7^^,^^10^ Their occurrences were the results of chromosome recombination in the cancer cells. They were the products of pathologic processes essential for cancer development. Normal tissues did not contain these chromosome features and, thus, did not express these transcripts. The detection of these fusion transcripts in the serum samples implies the presence of cancer cells. The widespread presence of these fusion transcripts in the serum samples from patients with HCC suggests that HCC cells shed these RNA fragments into the bloodstream. Possibly, the cancer cells also entered the blood circulation. Most patients with HCC achieved lower or even negative fusion-transcript levels after cancer treatment, suggesting that the cancer cells were the source of these fusion transcripts. Thus, detecting these fusion transcripts in the serum may have utility in screening for HCC and in assessing the cancer load. The machine-learning model tests developed from this study could be utilized in the following scenarios: i) to determine whether a liver biopsy is necessary in patients with a mass <LR5 or a nodule of unknown significance on liver imaging, ii) to evaluate cancer of the liver in patients with normal serum AFP levels, iii) to assess the efficacy of treatment and the presence of residual cancer in patients with HCC who have undergone treatment, and iv) as a routine screening test for early HCC in high-risk individuals before any expensive radiology imaging is performed.

The non-HCC cohort comprised individuals with other medical conditions (Supplemental Table S2). Most of them had liver disease. A large number of these individuals had pre-HCC conditions, including cirrhosis/fibrosis (29.3%, 22/75), nonalcoholic steatohepatitis/steatosis (32%, 24/75), hepatitis virus B/C infection (4%, 3/75), and alcoholic liver disease (2.6%, 2/75). A significant number of individuals from control groups were also positive for oncogenic fusion transcripts, such as *MAN2A1–FER*, *SLC45A2–AMACR*, and *PTEN–NOLC1*, albeit mostly at lower frequencies and not reaching the threshold of HCC determined by the machine-learning models. Many of these individuals had a history of other malignancies, suggesting that these fusion genes may come from other cancers.^11^^,^^37^^,^^38^ Yet, some individuals had no known history of malignancy but tested positive for these fusion transcripts. The cause of the presence of these fusion transcripts in these individuals' blood samples remained unclear. One speculation was that cancer cells shedding these fusion-transcript fragments were very dispersed and had not yet formed an imageable nodule. Alternatively, these fusion genes may be present in pre-malignant tissue, and the pre-malignant cells shed the fusion transcripts into the bloodstream. Regardless of either interpretation, clinical follow-up of these individuals might be warranted given that positive fusion transcripts in the blood indicated genome alterations in some somatic cells.

One interesting finding of this study was the dynamic changes in serum fusion-transcript levels after treatment. While in most individuals fusion transcripts dropped to undetectable levels after treatment, significant numbers of patients with HCC had only partially decreased serum fusion transcripts. In many cases, some fusion transcripts disappeared from the serum, while others remained unchanged. New fusion transcripts emerged in the serum after treatment in a few instances. All of these scenarios suggested the multiclonal and multifocal nature of the HCC. HCC may occur in multiple locations in the liver. Location-based treatment may eliminate most imageable cancer nodules but may leave the unidentified HCC nodules untreated. In addition, new cancer nodules might arise after treatment if a patient has a high-risk background for HCC. Given that each fusion gene results from a specific genome alteration, the fusion-gene patterns may reflect specific clonal origins of the cancers. As a result, active serum fusion-transcript surveillance may hold promise to be used to assess the impact of treatment and the heterogeneity of HCC in an accurate and timely manner.

## Disclosure Statement

The authors have submitted US patent application 63/603240, "Fusion Genes Associated with Hepatocellular Carcinoma and Related Methods," dated November 28, 2023.
